# GLP-1 receptor agonists-SGLT-2 inhibitors combination therapy and cardiovascular events after acute myocardial infarction: an observational study in patients with type 2 diabetes

**DOI:** 10.1186/s12933-023-02118-6

**Published:** 2024-01-06

**Authors:** Raffaele Marfella, Francesco Prattichizzo, Celestino Sardu, Pier Francesco Rambaldi, Carlo Fumagalli, Ludovica Vittoria Marfella, Rosalba La Grotta, Chiara Frigé, Valeria Pellegrini, Davide D’Andrea, Arturo Cesaro, Paolo Calabrò, Carmine Pizzi, Roberto Antonicelli, Antonio Ceriello, Ciro Mauro, Giuseppe Paolisso

**Affiliations:** 1 Department of Advanced Medical and Surgical Sciences, University of Campania “Luigi Vanvitelli”, Piazza Miraglia, 2, 80138 Naples, Italy; 2 IRCCS MultiMedica, Via Fantoli 16/15, 20138 Milan, Italy; 3 Department of Precision Medicine, The University of Campania “Luigi Vanvitelli”, Naples, Italy; 4 Department of Cardiology, Hospital Cardarelli, Naples, Italy; 5 Division of Clinical Cardiology, A.O.R.N. Sant’Anna e San Sebastiano’, University of Campania “Luigi Vanvitelli”, Naples, Italy; 6 Cardiology Unit, IRCCS Azienda Ospedaliera-Universitaria di Bologna, Bologna, Italy; 7 Department of Medical and Surgical Sciences-DIMEC-Alma Mater Studiorum, University of Bologna, Bologna, Italy; 8 Cardiology Unit, IRCCS INRCA, Ancona, Italy; 9 UniCAMILLUS, International Medical University, Rome, Italy

**Keywords:** SGLT-2 inhibitors, GLP-1 receptor agonists, MACE, Heart failure, Myocardial infarction, Glucose-lowering drugs, Combination therapies, Diabetes algorithm

## Abstract

**Background:**

Few studies explored the effect of the combination of glucose sodium-cotransporter-2 inhibitors (SGLT-2i) and glucagon-like peptide-1 receptor agonists (GLP-1RA) on the incidence of cardiovascular events in patients with type 2 diabetes (T2D) and acute myocardial infarction (AMI).

**Methods:**

We recruited patients with T2D and AMI undergoing percutaneous coronary intervention, treated with either SGLT-2i or GLP-1RA for at least 3 months before hospitalization. Subjects with HbA1c < 7% at admission were considered in good glycemic control and maintained the same glucose-lowering regimen, while those with poor glycemic control (HbA1c ≥ 7%), at admission or during follow-up, were prescribed either a SGLT-2i or a GLP-1RA to obtain a SGLT-2i/GLP-1RA combination therapy. The primary outcome was the incidence of major adverse cardiovascular events (MACE) defined as cardiovascular death, re-acute coronary syndrome, and heart failure related to AMI during a 2-year follow-up. After 3 months, the myocardial salvage index (MSI) was assessed by single-photon emission computed tomography.

**Findings:**

Of the 537 subjects screened, 443 completed the follow-up. Of these, 99 were treated with SGLT-2i, 130 with GLP-1RA, and 214 with their combination. The incidence of MACE was lower in the combination therapy group compared with both SGLT-2i and GLP-1RA treated patients, as assessed by multivariable Cox regression analysis adjusted for cardiovascular risk factors (HR = 0.154, 95% CI 0.038–0.622, P = 0.009 vs GLP-1RA and HR = 0.170, 95% CI 0.046–0.633, P = 0.008 vs SGLT-2i). The MSI and the proportion of patients with MSI > 50% was higher in the SGLT-2i/GLP-1RA group compared with both SGLT-2i and GLP-1RA groups.

**Interpretation:**

The combination of SGLT-2i and GLP-1RA is associated with a reduced incidence of cardiovascular events in patients with T2D and AMI compared with either drug used alone, with a significant effect also on peri-infarcted myocardial rescue in patients without a second event.

*Trial registraition* ClinicalTrials.gov ID: NCT06017544.

**Supplementary Information:**

The online version contains supplementary material available at 10.1186/s12933-023-02118-6.

## Introduction

Sodium–glucose cotransporter-2 inhibitors (SGLT-2i) and glucagon-like peptide-1 receptor agonists (GLP-1RA) have individually been shown to reduce the risk of major adverse cardiovascular events (MACE) in patients with type 2 diabetes (T2D) and established atherosclerotic cardiovascular disease (ASCVD) or multiple CVD risk factors [1, 2]. In particular, GLP-1RA consistently reduced atherosclerosis-related events, while SGLT-2i were demonstrated to attenutate also heart failure and kindey-related events, even in patients without T2D [3–5]. However, few patients enrolled in these clinical trials used the other drug as background therapy. Thus, the information relative to the efficacy of their combination are limited [6–8], particularly in patients with a recent acute myocardial infarction (AMI).

Previous studies evidenced that these drugs uniquely improved survival in patients with T2D and AMI [9–11]. As GLP-1RAs and SGLT-2i act with different mechanisms, resulting in a complementary pharmacodynamic amelioration of both atherosclerosis progression and heart failure development, their combination might improve outcomes of T2D patients with AMI [7, 12]. However, no study investigated the effect of the combination of GLP-1RA and SGLT-2i in people with T2D and an acute cardiovascular event on the incidence of MACE, nor on the degree of post-infarction myocardial rescue. Therefore, we conducted a prospective observational study in patients with T2D and hospitalized for AMI to evaluate the effect of GLP‑1RAs and SGLT-2i combination therapy on MACE, assessed as the cumulative incidence of all-cause mortality, acute coronary syndrome, and hospitalization for heart failure. After 3 months from AMI, we also assessed the myocardial salvage index (MSI), i.e. the difference between the actual and potential infarct size, the latter defined as the initial area at risk during acute coronary occlusion.

## Methods

### Study design and participants

We performed a multicentre, prospective observational study (ClinicalTrials.gov number, NCT06017544). Consecutive T2D patients with first AMI and treated with GLP-1 RA or SGLT-2i for at least 3 months prior to hospitalization were enlisted for this study. T2D patients without previous cardiovascular events hospitalized for AMI, either with ST-elevation myocardial infarction (STEMI) or non-ST-elevation myocardial infarction (NSTEMI) and referred for percutaneous coronary intervention (PCI) were screened from January 1, 2017, to December 31, 2021. Patients were stratified according to their glycemic control at the time of hospitalization [13]. As suggested by the ADA Standard of Care in diabetes [14], T2D patients with HbA1c < 7% were considered in good glycemic control and thus no drug was added to their glucose-lowering regimen and remained wither on GLP-1 RA or SGLT-2i. Individuals with poor glycemic control (HbA1c ≥ 7%) at the moment of admission or during follow-up were prescribed either an SGLT-2i or a GLP-1RA to receive a GLP-1RA/SGLT-2i combination therapy (Additional file 1: Figure S1). At admission, the therapy with sulphonylureas was discontinued [15].

Exclusion criteria were evidence of heart failure, valvular defects, malignant neoplasms, or secondary causes of hypertension. PCI was performed according to standard guidelines. Before PCI, all patients received loading doses of aspirin (300 mg) and a P2Y12 inhibitor (ticagrelor 180 mg; prasugrel 60 mg; or clopidogrel 300 to 600 mg). All patients underwent primary PCI within 3 h and received drug-eluting stents. After PCI, patients received lifelong aspirin plus a P2Y12 inhibitor for > 1 year, unless there was an unavoidable reason for stopping antiplatelet therapy.

Clinical variables were measured with standard procedures and angiographic data were also collected. Hypoglycemic episodes, i.e. any measurement of glycemia < 70 mg/dL, were self-reported by patients during follow-up visits. Medications such as renin–angiotensin–aldosterone system blockers, beta-blockers, and statins were prescribed according to guidelines [16]. After discharge from hospital, all patients were followed quarterly for two years after PCI as outpatients to maintain HbA1c level at < 7% and monitor weight and LDL cholesterol (Additional file 1: Table S1). During the follow-up, a multifactorial, person-centred approach was used to manage the glycemic, weight, and cardiorenal targets [14].

The myocardial rescue, assessed as MSI, was assessed by single-photon emission computed tomography (SPECT) before hospital discharge and after 3 months, as previously described [17]. MSI data were calculated as follows: AAR (%)-infarct size (%)/AAR. All SPECT analyses were performed by experienced radiologists blinded to patients’ and angiographic characteristics. Patients with incomplete data or lost during follow-up were excluded from the analysis. Sample size was selected based on previous studies with a similar design [18, 19].

### Study endpoints

The primary endpoint of the study was the composite of the incidence of all-cause mortality, hospitalization for heart failure, and acute coronary syndrome, such as STEMI, NSTEMI and unstable angina [16]. To avoid counfouning, only one event classification was allowed for each patients. Adjudication of events was perfomed by clinicians blinded to the study’s groups. The secondary endpoint was the proportion of patients with MSI values > 50% of the area at risk after 3 months in the three groups.

### Statistical analysis

Data were presented as numbers and frequencies for categorical variables and as median and interuartile range for continuous variables. The distribution of variables was assessed with the Shapiro–Wilk test. The differences between the three study groups were assessed using Kruskal–Wallis followed by Dunn’s test. Categorical variables were compared using χ^2^-test. Cox regression analysis was used to examine the association between the combination of GLP-1RA and SGLT-2i and the incidence of the composite outcome and was adjusted for age, sex, BMI, diabetes duration, glycemic control (admission, 3-months, 24-months HbA1c mean levels), LDL-cholesterol, triglycerides, troponin, creatinine, minimal lumen diameter (MLD), the prevalence of STEMI, hypertension, dyslipidemia and smoking. Multivariate logistic regression analyses were performed to estimate odds ratios (OR) and 95% confidence intervals (CI) for the MSI values > 50% of the area at risk and was adjusted for age, sex, BMI, diabetes duration, glycemic control, LDL-cholesterol, triglycerides, troponin, creatinine, MLD, the prevalence of STEMI, hypertension, dyslipidemia, and smoking. Two-sided P values < 0.05 were considered statistically significant. All calculations were performed using SPSS 29. Graphs were prepared using GraphPad Prism version 9.1.2.

## Results

### Study population

**Fig. 1:**
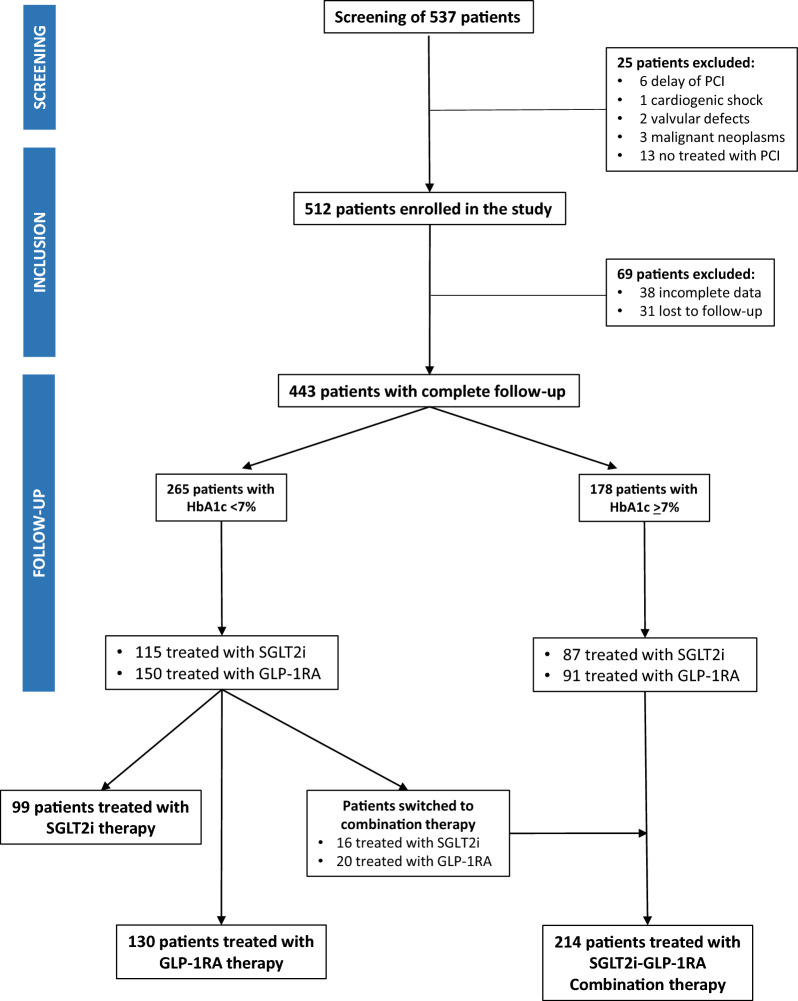
STROBE diagram showing the patients included in the different phases of the study

We screened 537 T2D individuals treated with GLP-1 RA or SGLT-2i, who underwent PCI for their first STEMI or NSTEMI. Of these, 443 completed the 24 months follow-up (Fig. 1). At admission, 265 patients were in good glycemic control (HbA1c = 6.8 ± 0.18%) and 178 were in poor glycemic control (HbA1c = 9.1 ± 1.5%). Among subjects with good glycemic control, 115 were treated with SGLT-2i (treatment duration = 12 ± 8 months) and 150 with GLP-1RA (treatment duration = 13 ± 3 months). All patients with poor glycemic control, initially treated with SGLT-2i (n = 87) or GLP-1RA (n = 91), were switched to combination therapy during their hospital stay. Among patients with good glycemic control, 16 treated with SGLT-2i and 20 treated with GLP-1RA were switched to combination therapy for HbA1c ≥ 7% during the follow-up. For these, the mean duration of treatment with the combination was 9.1 ± 3 2.5 months. Thus, the final study population included 99 patients treated with SGLT-2i therapy, 130 patients treated with GLP-1RA therapy, and 214 patients treated with the combination therapy (Fig. 1).

At baseline, there were no differences in sex distribution, smoking habits, hypertension, dyslipidemia, total cholesterol, LDL-cholesterol, HDL-cholesterol, troponin and creatinine levels among the groups. BMI was higher in SGLT-2i-treated individuals compared with both GLP-1RA and combination therapy patients while individual assigned to the combination therapy were older and had higher admission HbA1c, triglyceride levels, and diabetes duration (Table 1). Angiographic data showed that lesion length and reference diameter were similar between groups while the mean minimal luminal diameter (MLD) was lower in patients with poor glycemic control. After PCI, the MLD increased and was comparable in all groups. Moreover, all patients had similar post-PCI stenosis (Table 1). Fifteen SGLT-2i patients (15.1%), 21 GLP-1RA patients (16.1%), and 37 combination therapy patients (17.3%) were treated with triple antiplatelet treatment.

**Table 1:** Baseline characteristics

Variable	SGLT2i patients (n = 99)	GLP-1RA patients (n = 130)	SGLT2i + GLP-1RA patients (n = 214)	P-value
Age (years)	68 (63–73) Min = 57; Max = 76	67 (61–70) Min = 54; Max = 73	70 (64–75) Min = 55; Max = 79	**< 0.0001**
Male, n (%)	64 (64.6)	78 (60)	131 (61.2)	0.835
BMI (kg/m^2^)	28 (27–30) Min = 25; Max = 33	28 (27–29) Min = 15; Max = 31	28 (27–29) Min = 24; Max = 32	**0.005**
Diabetes duration (years)	15 (13–16.5) Min = 3; Max = 24	14 (13–16) Min = 3; Max = 24	15 (14–17) Min = 3; Max = 24	**0.0005**
Glucose (mg/dL)	137 (128–148) Min = 103; Max = 199	137 (128–155) Min = 99; Max = 234	189 (170.3–204) Min = 101; Max = 294	**< 0.0001**
HbA1c baseline	6.7 (6.5–6.8) Min = 6; Max = 9	6.7 (6.5–6.9) Min = 6.0; Max = 6.90	8.6 (7.8–9.6) Min = 6.4; Max = 13	**< 0.0001**
Total cholesterol (mg/dL)	211 (199–221) Min = 171; Max = 289	213 (199.8–222) Min = 82; Max = 274	213 (202–227) Min = 72; Max = 279	0.275
LDL cholesterol (mg/dL)	139 (124.3–148.6) Min = 7.2; Max = 200.6	134.9 (121–144) Min = 88.3; Max = 193.7	135.9 (122.1–146.6) Min = 85.6; Max = 216.55	0.157
HDL cholesterol (mg/dL)	38 (35–40) Min = 30; Max = 45	38 (36–40) Min = 30; Max = 45	38 (35–40) Min = 30; Max = 46	0.351
Triglycerides (mg/dL)	193 (183–208.5) Min = 159; Max = 265	193 (182–213) Min = 159; Max = 266	211 (197–224) Min = 163; Max = 293	**< 0.0001**
Creatinine (mg/dL)	1 (0.9–1.1) Min = 0.7; Max = 1.3	1 (1–1.1) Min = 0.1; Max = 1.30	1 (1–1.1) Min = 0.1; Max = 1.3	0.173
Troponin (ng/L)	14 (13.8–15) Min = 9; Max = 21	14.1 (13.7–15.8) Min = 12; Max = 18	14.2 (13.8–15.3) Min = 12; Max = 18	0.877
Lesion length (mm)	20.3 (19.3–22.3) Min = 17.3; Max = 25.3	20.3 (19.3–22.3) Min = 17.3; Max = 25.30	21.2 (19.3–22.3) Min = 16.2; Max = 27.3	0.855
Ref diameter	2.7 (2.5–2.9) Min = 1.84; Max = 4.74	2.8 (2.6–2.9) Min = 2.5; Max = 2.20	2.8 (2.6–2.9) Min = 2.3; Max = 3.2	0.736
MLD (mm)	1.2 (1.1–1.3) Min = 0.7; Max = 1.5	1.1 (1.1–1.2) Min = 0.80; Max = 1.6	1 (0.9–1.1) Min = 0.1; Max = 9	**< 0.0001**
Post-stent MLD (mm)	2.5 (2–2.7) Min = 1.7; Max = 3.5	2.6 (2–2.7) Min = 2.00; Max = 3.10	2.6 (2–2.7) Min = 2; Max = 3.2	0.273
STEMI, n (%)	41 (41.4)	15 (11.5)	90 (42.1)	0.990
Hypertension, n (%)	63 (63.6)	95 (73.1)	131 (61.2)	0.917
Dyslipidemia, n (%)	37 (37.4)	50 (38.5)	87 (40.7)	0.837
Smokers, n (%)	10 (10.1)	15 (11.5)	23 (10.7)	0.940
Metformin, n (%)	76 (76.8)	100 (76.9)	166 (77.6)	0.984
DPP-IV inhibitors, n (%)	9 (9)	6 (4.6)	23 (10.7)	0.141
Thiazolidinediones, n (%)	9 (9)	14 (10.7)	20 (9.3)	0.889
Beta-blockers, n (%)	52 (52.5)	68 (52.3)	108 (50.5)	0.920
ARBs, n (%)	39 (39.4)	50 (38.5)	81 (37.9)	0.966
ACE inhibitors, n (%)	42 (42.4)	55 (42.3)	93 (43.5)	0.973
Calcium channel blockers, n (%)	31 (31.3)	44 (33.8)	68 (31.8)	0.899
Statin, n (%)	60 (60.6)	79 (60.8)	133 (62.1)	0.952
Diuretics, n (%)	26 (26.3)	35 (26.9)	62 (29)	0.855
Insulin, n (%)	35 (35.4)	48 (36.9)	81 (37.9)	0.913
Antiplatelet drugs, n (%)	64 (64.6)	82 (63.1)	133 (62.1)	0.879
Anticoagulant drugs, n (%)	22 (22.2)	22 (16.9)	77 (36)	**0.0003**
One-vessel disease, n (%)	38 (38.4)	50 (38.5)	78 (36.4)	0.912
Two-vessel disease, n (%)	49 (49.5)	62 (47.7)	107 (50)	0.916
Three-vessel disease, n (%)	11 (11.1)	20 (15.4)	23 (10.7)	0.414

For continuous variables, differences between the three groups were evaluated with the Kruskal–Wallis test and data are expressed as median (Q1–Q3). For categorical variables, intergroup differences were analyzed using the Chi-square test. For continuous variables, also the minimum and maximum values are shown. Significant p values are highlighted in bold

### Glycemic control, weight, and LDL cholesterol during follow-up

**Fig. 2:**
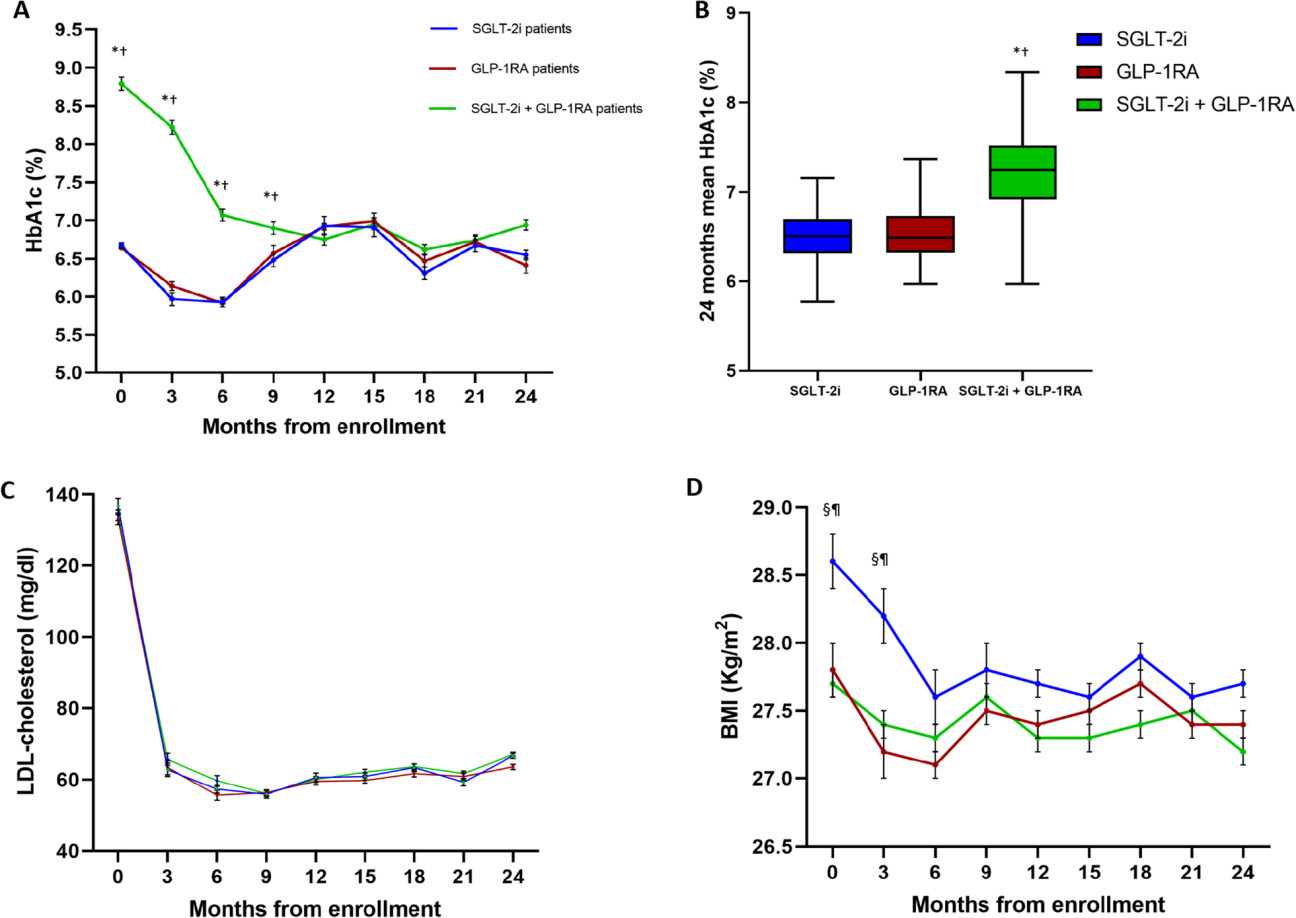
Trend of HbA1c, LDL-cholesterol, and BMI in the three groups during follow-up. Graphs showing the mean values of glycated haemoglobin (HbA1c) (**A**), LDL-cholesterol (**C**), and BMI (**D**), along with the mean cumulative HbA1c (**B**), in the three groups during the successive visits of the follow-up. Error bars are ± SE for panels **A**, **C**, and **D**. *P < 0.05 SGLT-2i/GLP-1RA vs SGLT-2i; ^†^P < 0.05 SGLT-2i/GLP-1RA vs GLP-1RA; ^⁋^P < 0.05 SGLT-2i vs SGLT-2i/GLP-1RA; ^§^P < 0.05 SGLT-2i vs GLP-1RA; Kruskal–Wallis followed by Dunn’s test for all. Boxplots show the median, 25th, and 75th percentiles, range, and extreme values

During the follow-up, the combination therapy group had higher 24-month mean HbA1c levels compared with patients in either the GLP-1RA or SGLT-2i group, a difference driven by the poorer glycemic control in the first 9 months after AMI (Fig. 2A, B). There were no differences in annual-incidence rate of hypoglycemic events among the groups: 2 SGLT-2i patients (2.0%), 3 GLP-1RA patients (2.3%), and 5 combination therapy patients (2.3%) (data not shown). LDL-cholesterol decreased in all patients without differences among groups (Fig. 2C). After 6 months, the BMI, initially higher in SGLT-2i patients, decreased without significant differences between the groups (Fig. 2D).

### Primary endpoint and myocardial salvage index

**Fig. 3:**
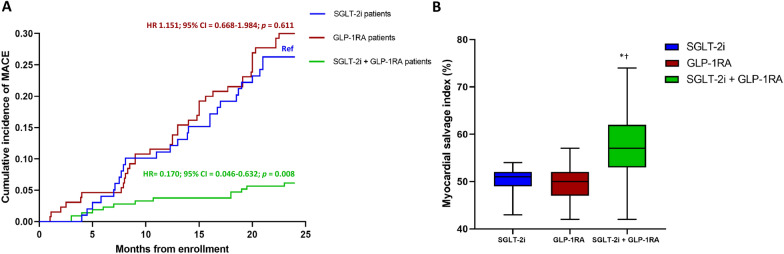
Primary endpoint and myocardial salvage index. Cumulative incidence of MACE in the groups on therapy with SGLT-2i (ref), GLP-1RA, and their combination with the relative hazard ratios (HR) and confidence intervals (CI) resulting from the relative Cox regression analysis (**A**); percentange of myocardial salvage index assessed thorugh SPECT after 3 months in the three groups (**B**). *P < 0.05 SGLT-2i/GLP-1RA vs SGLT-2i; ^†^P < 0.05 SGLT-2i/GLP-1RA vs GLP-1RA; Kruskal–Wallis followed by Dunn’s test. Boxplots show the median, 25th, and 75th percentiles, range, and extreme values

The the primary endpoint occurred in 26 individuals (26.3%) in the SGLT-2i group, 39 (30%) in the GLP-1RA group, and 13 (6.1%) in the GLP-1RA-SGLT-2i combination therapy group. The incidence of MACE was lower in the combination therapy group compared with both GLP-1RA and SGLT-2i treated subjects (HR = 0.154, 95% CI 0.038–0.622, P = 0.009 vs GLP-1RA and HR = 0.170, 95% CI 0.046–0.633, P = 0.008 vs SGLT-2i), as assessed by multivariable Cox regression analysis adjusted for age, sex, BMI, diabetes duration, glycemic control (admission, 3-months, 24-months HbA1c mean levels), LDL-cholesterol, triglycerides, troponin, creatinine, MLD, the prevalence of STEMI, hypertension, dyslipidemia and smoking (Fig. 3A and Additional file 1: Table S2). Exploration of the incidence on individual outcomes of the composite endpoint in the three groups evidenced that the effect was driven by the benefit provided by the combination compared with SGLT-2i alone on the incidence of acute coronary syndrome and by the reduction in the incidence of hospitalization for heart failure observed with the combination compared with GLP-1RA alone (Additional file 1: Table S3).

The MSI assessment, performed after 3 months, showed that the amount of survived peri-infarcted myocardium was higher in patients treated with the GLP-1RA-SGLT-2i combination therapy compared with both the patients treated with SGLT-2i or GLP-1RA (Fig. 3B). The odds of having an MSI > 50% of the area at risk in patients treated with combination therapy were greater compared with the other two groups (OR = 4.06, 95% CI 2.40–6.85; P < 0.0001 vs SGLT-2i; OR = 7.51, 95% CI 4.59–12.29; P < 0.0001 vs GLP-1RA).

## Discussion

In this prospective observational study, SGLT-2i/GLP-1RA combination therapy was associated with a lower incidence of a composite of acute coronary syndrome, hospitalization for heart failure, and all-cause mortality, compared with either SGLT-2i or GLP-1RA therapies used alone in patients with T2D and a recent AMI. The effect was independent of glycemic control, including progressively lower HbA1c values during follow-up, thus suggesting that the benefit observed with the combination therapy is unlikely to be ascribable to an improvement of glycemic control derived from the add-on of a further glucose-lowering drug.

Few studies explored the effect of the combination of SGLT-2i and GLP-1RA on CVD. Data from a post-hoc analysis of the AMPLITUDE-O trial evidenced that the effect of efpeglenatide, a GLP-1RA, on large range of cardiovascular and renal endpoints was not influenced by the baseline use of SGLT-2i [7]. However, few patients were on this background therapy and the trial was not designed to test the effect of this combination. A registry-based study explored specifically the effect of the combined treatment with SGLT-2i and GLP-1RA on cardiovascular outcomes in subjects with T2D and no previous CVD. Compared with other combination regimens, SGLT-2i-GLP-1RA combination reduced the incidence of MACE and of heart failure in individuals in primary prevention [8]. Another study using registry-derived data showed that, compared with the initiation of sulfonylurea, the addition of SGLT-2i to GLP-1RA therapy was associated with a significantly lower incidence of MACE, driven by a reduction in the incidence of myocardial infarction and mortality, but also of hospitalizations for heart failure [9]. Subgroup analyses from this same study evidenced that the prevalence of CVD at baseline did not affect the results. However, given the nature of their designs, none of these studies explored specifically the effect of the SGLT-2i-GLP-1RA combination in patients with T2D hospitalized for AMI. Here, we extend previous observations by showing that when these drugs are used in combination, they are associated with a reduction in the incidence of a composite of acute coronary syndrome, hospitalization for heart failure, and all-cause mortality compared with either drug used alone. Specifically, the benefit seems provided by the effects of SGLT-2i on hospitalization for heart failure and of GLP-1RA on acute coronary syndrome, two observations in line with existing knowledge [4–6]. On the other hand, our study was not powered to detect differences in individual outcomes and thus the relative results should be interpreted with caution.

SGLT-2i-GLP-1RA combination was also associated with an improved rescue of peri-infarcted myocardium independently of glycemic control, as evidenced by the MSI after 3 months of follow-up. Previous studies showed that in patients with AMI and T2D, both SGLT-2i and GLP-1RA reduced infarct size and peri-infarct tissue inflammation [17] and prevented also the loss of pump function assessed as left ventricular ejection fraction [20]. Data presented here might suggest that SGLT-2i and GLP-1RA synergize to improve post-AMI remodelling. Mechanistically, a large range of metabolic, molecular, and hemodynamic phenomena have been proposed to explain the benefit provided by both these drugs [21–23]. Similarly, a plethora of intermediate risk factors, e.g*.* body weight, glycemic control, and blood pressure, are improved by both these classes and are further reduced when they are used in combination [24]. Of note, the difference in the incidence of MACE between the three groups is consistent with early separation between the relative curves, suggesting a combinatorial benefit rather than simply additive. In support of this postulate, our data evidenced that the protective cardiovascular effects of the combination therapy were independent of glycemic, weight, and blood pressure controls. Thus, the outcomes observed with the combination therapy might be linked to a putative synergic molecular effect on the heart and the vasculature through improving a range of metabolic, anti-inflammatory, and regenerative pathways [25–28]. Which, if any, of the canonical and non-canonical mechanisms proposed underly the observations provided here is unkown and should be explored by future studies.

Limitations of this study includes its observational nature. Patients were not randomized and residual confounders cannot be excluded. In addition, given the study design, we compared a combined regimen with single drugs. Moreover, our sample size was not adequate to explore the effect of the combination therapy on individual outcomes. For the same reason, no subgroup analysis, e.g. stratification for sex or diabetes duration, was planned. Finally, we did not include stroke in the composite primary outcome. However, our study population included patients with low baseline risk factors for stroke, such as the absence of atrial fibrillation, and the absence of significant carotid artery disease. In addition, our study had a relatively short follow-up duration. Future studies of longer duration and larger sample sizes should explore the effect of the combination therapy on this endpoint.

In summary, the use of SGLT-2i-GLP-1RA combination therapy was associated with a reduced incidence of cardiovascular events in T2D patients hospitalized for AMI compared with either drug used alone. These and other data might prompt the design of trials using these agents in combination, in order to substantiate the possible incremental efficacy of this combination regimen to ameliorate the noxious effect of T2D on CVD.

### Supplementary Information

**Additional file 1: Figure S1.** Study design. **Table S1.** Scheme of visits. **Table S2.** Results of the Cox regression analysis. **Table S3.** Individual endpoints of the composite outcome.

## Data Availability

Data are available at request.
