# Supplementary material for: GLP-1 receptor agonists-SGLT-2 inhibitors combination therapy and cardiovascular events after acute myocardial infarction: an observational study in patients with type 2 diabetes
Source: Cardiovasc Diabetol. 2024 Jan 6;23:10. doi: 10.1186/s12933-023-02118-6 (PMC10771648; doi:10.1186/s12933-023-02118-6)
Supplement: Supplementary file 1 — Additional file 1: Figure S1. Study design. Table S1. Scheme of visits. Table S2. Results of the Cox regression analysis. Table S3. Individual endpoints of the composite outcome. [file 12933_2023_2118_MOESM1_ESM.docx]

**ADDITIONAL APPENDIX**

Supplement to: Marfella R, Prattichizzo F, Sardu C, et al. GLP-1 Receptor Agonists-SGLT2 Inhibitors Combination Therapy and Cardiovascular Events After Acute Myocardial Infarction: an Observational Study in Patients with Type 2 Diabetes

Marfella et. al

**TABLE OF CONTENTS**

1. Figure S1. Study design……………………………………………………..1
2. Table S1. Scheme of visits…...………………………………………….......2
3. Table S2. Results of the Cox regression analysis……………………….…...3
4. Table S3. Individual endpoints of the composite outcome…………….…….4

**Figure S1.** **Design of the study.**

**
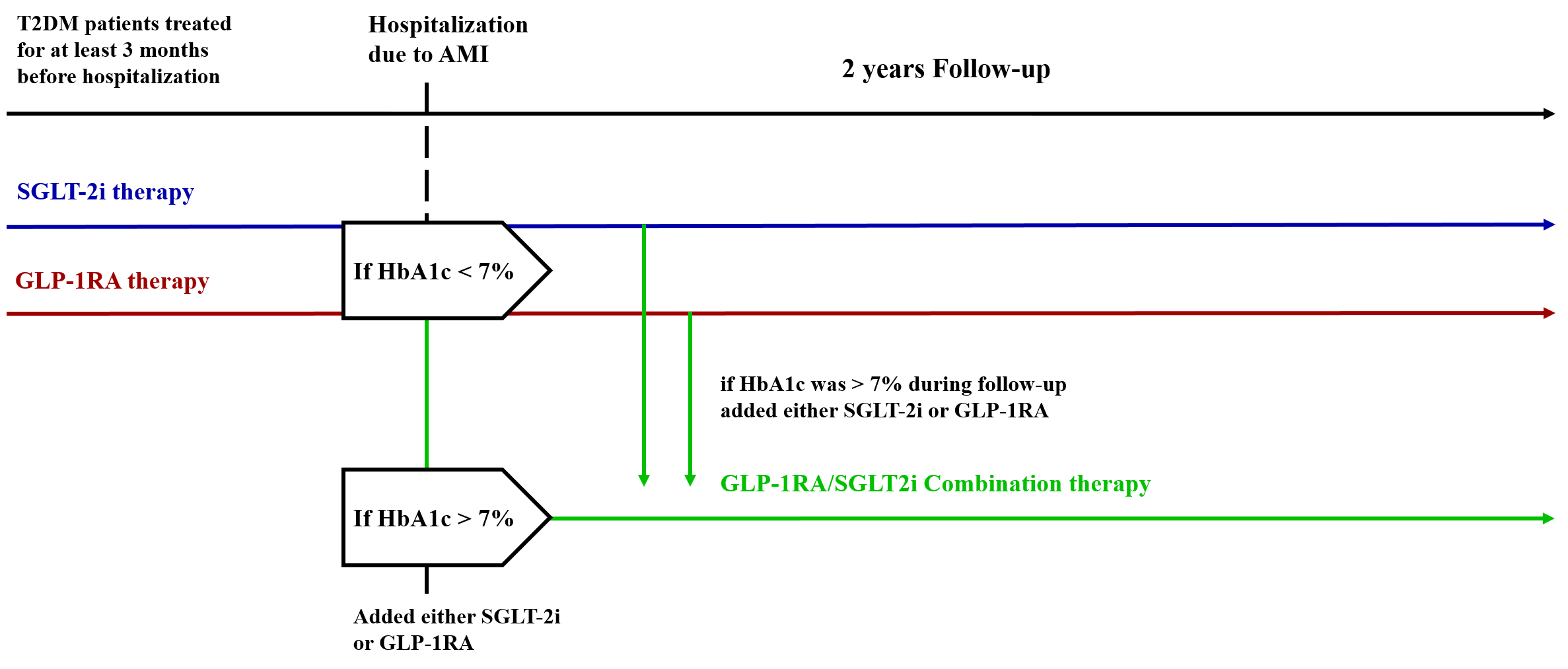
**

**Table S1.** **Scheme of visits during follow-up.**

**2 years follow-up**

| ***Variables*** | **Baseline** | **3 months** | **6 months** | **9 months** | **12 months** | **15 months** | **18 months** | **21 months** | **24 months** |
| --- | --- | --- | --- | --- | --- | --- | --- | --- | --- |
| ***Age*** | **X** |  |  |  |  |  |  |  |  |
| ***Sex*** | **X** |  |  |  |  |  |  |  |  |
| ***Clinical history*** | **X** |  |  |  |  |  |  |  |  |
| ***Pharmacological therapies*** | **X** | **X** | **X** | **X** | **X** | **X** | **X** | **X** | **X** |
| ***BMI*** | **X** | **X** | **X** | **X** | **X** | **X** | **X** | **X** | **X** |
| ***HbA1c*** | **X** | **X** | **X** | **X** | **X** | **X** | **X** | **X** | **X** |
| ***Lipid profile*** | **X** | **X** | **X** | **X** | **X** | **X** | **X** | **X** | **X** |
| ***Glucose*** | **X** |  |  |  |  |  |  |  |  |
| ***Creatinine*** | **X** |  |  |  |  |  |  |  |  |
| ***Troponin*** | **X** |  |  |  |  |  |  |  |  |
| ***Lesion length*** | **X** |  |  |  |  |  |  |  |  |
| ***Ref diameter*** | **X** |  |  |  |  |  |  |  |  |
| ***MLD*** | **X** |  |  |  |  |  |  |  |  |
| ***Post-stent MLD*** | **X** |  |  |  |  |  |  |  |  |
| ***Other drugs*** | **X** |  |  |  |  |  |  |  |  |
| ***Single-photon emission computed tomography*** | **X** | **X** |  |  |  |  |  |  |  |

**Table S2. Results of the Cox regression analysis exploring the incidence of MACE in the three groups.**

|  |  |  | |  |
| --- | --- | --- | --- | --- |
| **Variable** | **HR** | **Lower** | **Higher** | ***p*** |
| SGLT-2i | REF | REF | REF |  |
| GLP-1RA | 1,151 | 0,668 | 1,984 | 0,611 |
| SGLT-2i + GLP-1RA | 0,170 | 0,046 | 0,632 | 0,008 |
| Age | 0,956 | 0,913 | 1,001 | 0,056 |
| Sex | 0,914 | 0,545 | 1,531 | 0,732 |
| BMI | 0,979 | 0,856 | 1,119 | 0,753 |
| Diabetes duration | 1,050 | 0,976 | 1,129 | 0,193 |
| HbA1c during admission | 1,114 | 0,737 | 1,684 | 0,610 |
| HbA1c after 3 months | 1,227 | 0,914 | 1,648 | 0,173 |
| HbA1c follow-up mean | 0,494 | 0,223 | 1,096 | 0,083 |
| MLD | 0,708 | 0,141 | 3,548 | 0,675 |
| STEMI | 1,223 | 0,768 | 1,948 | 0,396 |
| Hypertension | 1,007 | 0,616 | 1,645 | 0,979 |
| Dyslipidemia | 0,660 | 0,399 | 1,092 | 0,106 |
| LDL-cholesterol | 1,009 | 0,997 | 1,021 | 0,155 |
| One-vessel disease | 2,983 | 0,865 | 10,291 | 0,084 |
| Two-vessel disease | 2,648 | 0,758 | 9,248 | 0,127 |
| Three-vessel disease | 1,313 | 0,311 | 5,551 | 0,711 |
| Creatinine | 1,031 | 0,149 | 7,125 | 0,975 |
| Lesion length | 0,952 | 0,835 | 1,086 | 0,465 |
| Ref diameter | 0,543 | 0,240 | 1,227 | 0,142 |

HR= Hazard ratio; CI= confidence interval; BMI= Body mass index; HbA1c= glycated hemoglobin MLD= Mean lumen diameter

**95,0% CI**

**Table S3. Crude number of events for each component of the composite outcome and the relative unadjusted and adjusted hazard ratios.**

| **Outcomes** | **SGLT2i  patients**  **(n=99)** | **GLP-1RA  patients**  **(n=130)** | **SGLT2i + GLP-1RA patients (n=214)** | **Unadjusted HR (95.0% CI) combination vs SGLT2i** | **Adjusted* HR (95.0% CI) combination vs SGLT2i** | **Unadjusted HR (95.0% CI) combination vs GLP-1RA** | **Adjusted* HR (95.0% CI)  combination vs GLP-1RA** |
| --- | --- | --- | --- | --- | --- | --- | --- |
|  |  |  |  |  |  |  |  |
| **All-cause mortality. n (%)** | 7 (7.1) | 7 (5.4) | 3 (1.4) | 0.162 (0.042-0.618);  *p*=0.008 | 0.126 (0.005-3.095);  *p=*0.204 | 0.249 (0.069-0.897);  *p=*0.033 | 0.124 (0.003-5.863);  *p=*0.289 |
| **Heart failure. n (%)** | 4 (4.0) | 12 (9.2) | 5 (2.3) | 0.551 (0.135-2.247);  *p=*0.406 | 1.846 (0.094-6.161);  *p=*0.686 | 0.240 (0.090-0.643);  *p=*0.005 | 0.081 (0.008-0.842);  *p=*0.035 |
| **Acute coronary syndrome. n (%)** | 15 (15.2) | 20 (15.4) | 5 (2.3) | 0.1 (0.038-0.266);  *p=*<0.0001 | 0.020 (0.001-0.420);  *p=*0.012 | 0.131 (0.057-0.301);  *p=*<0.0001 | 0.092 (0.007-1.156);  *p=*0.065 |

*** Adjusted for age, sex, BMI, diabetes duration, glycemic control (admission, 3-months, 24-months HbA1c mean levels), LDL-cholesterol, triglycerides, troponin, creatinine, minimal lumen diameter (MLD), the prevalence of STEMI, hypertension, dyslipidemia and smoking.**
